# Study on the Ultimate Load Capacity of Cu-Ni Alloy Pipelines with Double Pitting Defects

**DOI:** 10.3390/ma19010121

**Published:** 2025-12-29

**Authors:** Xinglong Pan, Jianggui Han, Wenyong Guo, Hantao Chen, Yan Zeng, Zhe Wu, Li Yu, Liangwu Yu

**Affiliations:** Naval University of Engineering, Wuhan 430033, China; paxilo619@163.com (X.P.); hanjianggui@hotmail.com (J.H.); guowy202@163.com (W.G.); 1920191077@nue.edu.cn (H.C.); zengyan@whut.edu.cn (Y.Z.); 1920191013@nue.edu.cn (Z.W.)

**Keywords:** Cu-Ni alloy pipeline, double pitting defect, ultimate load capacity, finite element simulation, hydrostatic burst test

## Abstract

To accurately evaluate the load-bearing capacity of Cu-Ni alloy pipelines with double pitting corrosion defects, in this study, the influence of dual-defect morphological parameters on the ultimate load capacity was investigated through finite element simulation. On the basis of the ultimate load capacity model for single pit defects and simulation results, an assessment model was developed for Cu-Ni alloy pipelines containing double pitting defects, and its accuracy was validated through hydrostatic burst tests. The results indicate that the ultimate load capacity increases gradually with increasing interdefect center distance, asymptotically approaching the load-bearing capacity of a single-defect pipeline with an equivalent depth and diameter. The proposed model demonstrates excellent predictive performance, with a maximum error margin within 3%. During failure, Cu-Ni alloy pipelines with double pitting defects develop fine axial cracks at the defect sites while exhibiting significant overall bulging deformation. These findings can be effectively applied to predict the ultimate load capacity of Cu-Ni alloy pipelines with double pitting defects, providing substantial engineering value for an accurate assessment of the load-bearing capacity of pipelines with actual corrosion defects.

## 1. Introduction

Shipboard seawater pipelines operate in highly corrosive environments and are susceptible to severe corrosion degradation [1]. To mitigate this issue, copper-nickel (Cu-Ni) alloys with superior corrosion resistance are extensively utilized in modern seawater piping systems. Nevertheless, corrosion cannot be entirely eliminated. Corrosion-induced defects significantly compromise the load-bearing capacity of pipelines. Once the operating pressure surpasses this limit, pipeline failure is likely. This could cascade into failures of the main engine and related auxiliary equipment [2], thereby jeopardizing the vessel’s operational availability and mission readiness.

Given the severe consequences of pipeline failure, conducting rigorous ultimate load capacity assessments for corroded seawater pipelines is imperative for ensuring equipment safety and vessel navigation security [3]. While assessment methodologies for defective onshore oil and gas pipelines are well-established, supported by decades of global research involving theoretical modeling and full-scale burst tests, which have yielded a comprehensive body of standards and specifications [4,5,6,7], such established frameworks are not directly transferable to the more complex marine piping environment.

In contrast, mature assessment methodologies and standardized procedures for evaluating the load-bearing capacity of marine seawater pipelines, which are primarily constructed from copper-nickel alloys, remain underdeveloped. Corrosion in such pipelines typically manifests as colony (or clustered) corrosion, which can be discretized into individual corrosion pits. The mechanical interaction between adjacent defects leads to failure mechanisms that are fundamentally distinct from those in pipelines with single pit defects, thereby necessitating dedicated predictive approaches for assessing ultimate load capacity.

The investigation of the ultimate load capacity for colony corrosion defects aims to address two critical research questions:

(1) Determining the critical axial/circumferential spacing between double defects beyond which their interaction ceases and the load capacity converges to that of a single defect of equivalent size. This defines the minimum non-interaction distance.
(2) Establishing a quantitative mathematical model that characterizes the reduction in ultimate load capacity as a function of the center-to-center distance within the interaction zone, where mutual interference between defects is significant.

Substantial research progress has been made on the first issue over the past three decades. Studies on pipelines containing double pitting defects, dual localized uniform corrosion defects, and axial double groove-type defects [8,9,10,11] have systematically investigated the influence of defect morphology and spacing on ultimate load capacity. These studies consistently demonstrate that interaction effects intensify with increasing defect depth. Furthermore, based on research findings [12,13,14,15] and the DNV-RP-F101 standard [16], three distinct criteria have been summarized to determine whether closely spaced defects should be considered as interacting or independent. These criteria define threshold spacing limits: defects separated by distances greater than these thresholds can be regarded as independent, whereas those within the limits exhibit significant interaction.

Considerable work has also addressed the second issue. For instance, studies [17,18] employed extensive finite element analysis of thin-walled pipelines with axial double groove defects. Their work aimed to characterize the nonlinear relationship between defect spacing and ultimate capacity via linear approximations, yet proved insufficient for accurate modeling. In contrast, study [4] introduced a simplified equivalence method, treating complex multi-defect configurations as equivalent single defects. Here, the maximum local depth serves as the equivalent corrosion depth, and the overall defect envelope provides the equivalent length and width. While this offers a tractable engineering solution, it inherently neglects the load-carrying contribution of the intact material between defects, thereby yielding conservative assessments.

The DNV-RP-F101 standard [16] advanced this methodology by introducing defect projection techniques, thereby incorporating the effect of axial uncorroded ligaments between interacting defects. However, it does not account for circumferential uncorroded regions, which sustains its conservative nature and inherent limitations. To address these shortcomings, study [19] developed a computational framework for symmetric interacting corrosion that accommodates diverse defect typologies. More recently, the field has seen a pronounced shift toward data-driven approaches, with neural networks and machine learning models, often coupled with optimization algorithms, being widely employed to predict the burst pressure of corroded pipelines [20,21,22]. These emerging techniques offer novel perspectives and methodological pathways for assessing the residual load-bearing capacity of pipelines with corrosion defects.

Two principal methodologies exist for addressing the second issue: one simplifies the problem by treating multiple defects as an equivalent single defect, and the other seeks to directly establish a functional relationship between defect spacing and ultimate load capacity. The former approach often suffers from limited accuracy, primarily because it neglects the interaction effects between defects. The latter, while offering the potential for greater precision, is constrained by its reliance on abstract and often complex models. Although computational methods derived from such models generally outperform the simplified equivalent-defect approach in terms of accuracy, their complexity remains a barrier. Consequently, this study aims to identify an appropriate nonlinear function that accurately characterizes the relationship between defect spacing and ultimate load capacity. By fitting this function to data on center-to-center distance versus capacity, a more precise evaluation model for pipelines with axial double pitting defects will be developed, providing a foundation for further theoretical extension and practical application.

## 2. Materials and Research Methods

### 2.1. Test Materials

Cu-Ni alloy pipes are widely employed in marine environments owing to their excellent physical and mechanical properties, coupled with outstanding corrosion resistance and anti-fouling capabilities [23,24,25,26,27]. This proven reliability in long-term service has established them as a preferred material for seawater pipelines. Consequently, superior corrosion-resistant copper-nickel alloys have been increasingly adopted in modern marine piping systems, replacing many conventional materials. However, corrosion remains an inherent and unresolved challenge, even for this advanced material. Therefore, this study focuses on Cu-Ni alloy seawater pipelines to develop a predictive model for the ultimate load capacity of pipelines with interacting double pitting defects.

The mechanical properties of the Cu-Ni alloy (B10) were characterized via tensile testing performed on an INSTRON 5587 300 kN universal testing machine (manufactured by Instron, Norwood, MA, USA), following the GB/T 228.1-2010 standard [28]. The obtained data are summarized in Table 1.

### 2.2. Research Methods

**Figure 1:**
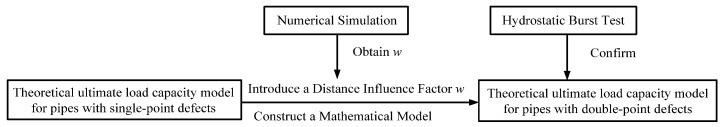
Research methodology and approach.

The ultimate load capacity assessment model for marine seawater pipelines is grounded in elastoplastic fracture mechanics, integrating the influence of pipeline material properties, defect morphology, and loading conditions. To develop and calibrate this model, a multi-stage methodology was implemented. First, the dynamic explicit finite element method was employed to perform numerical simulations of the ultimate load capacity of corroded pipelines, with the results used to calibrate and refine the initial theoretical model. Subsequently, the accuracy of the revised model was validated experimentally via hydrostatic burst tests. Collectively, these steps constitute an integrated research framework of “theoretical modeling, numerical simulation, experimental validation, and model refinement” to systematically evaluate the ultimate bearing capacity of pipelines with double pitting defects. The complete research methodology is illustrated in Figure 1.

To extend the theoretical model for pipelines with single pits to the double-defect case, a spacing influence factor (*w*) was introduced to quantify the interaction between defects, leading to a new theoretical framework for ultimate bearing capacity. This model, along with its underlying numerical simulations, was subsequently validated through physical experiments. The factor *w* itself was derived from finite element analyses. Furthermore, by developing a parameterized finite element model, a systematic parametric study was conducted to investigate how *w* varies with defect size, center-to-center distance, and depth. The results provided essential parameter inputs for the formulation of the theoretical model.

#### 2.2.1. Simulation Method

The dynamic explicit finite element method employs an explicit solution scheme and is typically used to calculate transient impact responses in high-speed structures and to simulate material stress degradation and failure. The simulations in this study meet the industry quasi-static criterion (kinetic energy/internal energy ratio < 5%) to address a quasi-static material failure problem using the Abaqus/Explicit (2016) module.

The double pitting defects investigated in this section are characterized using the dimensionless parameters of the corrosion depth ratio *N* and corrosion diameter $D_{P}$ to quantify the corrosion severity. The ultimate load capacity of Cu-Ni alloy pipelines with these defects at varying distances was calculated using the finite element method. The double pitting defects consist of two defects with equal diameter and depth; thus, the defect parameters include the corrosion depth ratio *N*, the corrosion diameter $D_{P}$, and the center distance $l_{d}$ between the axial double pitting defects.

**Figure 2:**
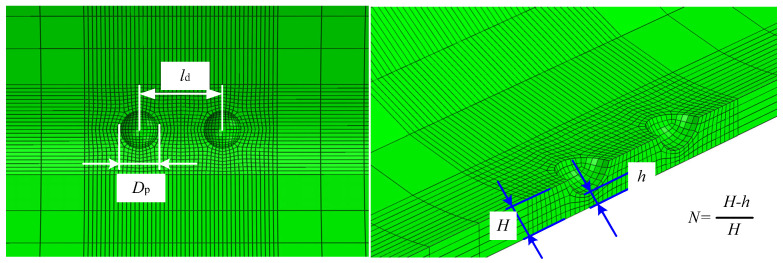
Schematic diagram of defect parameter definitions.

The defect morphology adopted in this study is modeled as a spherical cap. Geometrically, the pipeline’s inner surface is treated as a reference plane. A regular spherical cap, representing localized material loss, is then superimposed along the wall-thickness direction. This is conceptually equivalent to intersecting a sphere with the inner surface as the cutting plane; the resulting spherical cap defines the defect geometry. The intersection of this cap with the inner surface forms a circle with a diameter defined as $D_{P}$. The depth ratio, *N*, is defined as the height of the spherical cap divided by the intact wall thickness of the pipeline. For double pitting defects, the model considers two such spherical caps with identical geometry aligned along the same axis. The distance between their centers is denoted as $l_{d}$. These geometric relationships are summarized in Figure 2.

The assumptions and definitions outlined above are reasonable. The investigation focuses on a Cu-Ni alloy pipeline of grade BFe10-1.6-1 (commonly designated as B10 cupronickel, with 10% Ni content). The homogeneity assumption is justified by the standardized production and manufacturing processes of this material. Furthermore, the simplification of irregular natural corrosion into regularized geometries, such as hemispheres, semi-ellipsoids, or cylinders, aligns with established engineering practices codified in international standards like DNV-RP-F101 [16] and API 579 [7].

The limitation of the model is that real corrosion defects can be more complex, particularly after prolonged exposure to corrosive environments. Nevertheless, the adopted simplifications are both necessary and justified, as they ensure the model remains tractable and widely applicable. Consequently, the outcomes of this study offer tangible practical value for engineering-based safety assessments and residual-strength predictions of corroded pipelines.

This study investigates the relationship between the ultimate load capacity and the center-to-center distance of axially aligned double pitting defects under two distinct parametric configurations.

Condition 1: Constant pitting diameter with varying depth. Details of the pipe samples are provided in Table 2. Twenty-eight pipelines (AP1#–AP28#) were divided into four groups. All samples had a fixed pitting diameter ($D_{P}$ = 12 mm), with the corrosion depth ratio (*N*) set to 0.2, 0.4, 0.6, and 0.8, respectively.

Condition 2: Constant corrosion depth ratio with varying diameter. Sample details are given in Table 3. Twenty-one pipelines (AP29#–AP49#) were divided into three groups, all with a fixed depth ratio (*N* = 0.8). The pitting diameter ($D_{P}$) was set to 6, 9, and 15 mm, respectively. To ensure parametric continuity, the data for $D_{P}$ = 12 mm were sourced from the corresponding samples under Condition 1 (AP4#, AP8#, AP12#, AP16#, AP20#, AP24#, AP28#).

The simulations for all 49 pipelines listed in Table 2 and Table 3 were completed using the explicit finite element method. Preliminary calculations indicated that for the Cu-Ni alloy pipeline with a diameter of 90 mm and wall thickness of 5 mm used in this study, the influence of the center distance of the axial double pitting defect on the ultimate load capacity stabilized when the distance exceeded 40 mm. The minimum center distance was set as the sum of the radii of the two defects, considering that the interaction effect strengthens as the defects approach each other. Consequently, the center distance range was established from 13 to 40 mm. A denser sampling interval was adopted at smaller center distances where the gradient of influence is more pronounced, whereas a sparser interval was used at larger distances where the variation becomes more gradual.

Both ends of the pipeline were fully constrained, and a linearly increasing pressure was applied to the entire inner wall surface, including the defect regions. This configuration replicates the actual boundary and loading conditions of shipboard pipelines. The true stress–strain data from tensile tests of the Cu-Ni alloy were incorporated into the model to account for the intense localized plastic deformation at the defects prior to fracture, thereby capturing the significant material and geometric nonlinearity exhibited during deformation. The quasi-static process was simulated with the Abaqus/Explicit solver. Stress and deformation characteristics under internal pressure were evaluated based on a ductile damage criterion. Finally, a comprehensive simulation matrix encompassing all 49 pipeline models with varying defect types and parameters was established.

#### 2.2.2. Test Method

Axial double pitting defects commonly occur in pipelines subjected to cavitation corrosion. Under the repeated impact of collapsing bubbles, the metal on the inner surface of the pipeline spalls off, forming spherical pits that typically appear as corrosion colonies. For modeling purposes, this scenario is simplified by considering only two defect pits arranged axially.

Electrical discharge machining was employed to simulate axial double pitting defects by ablating the metal using a CNC electric discharge machine (model CNC-850). This method provides a fine surface finish and excellent control over the defect geometry during processing, and maintains machining errors within 0.1 mm.

**Figure 3:**
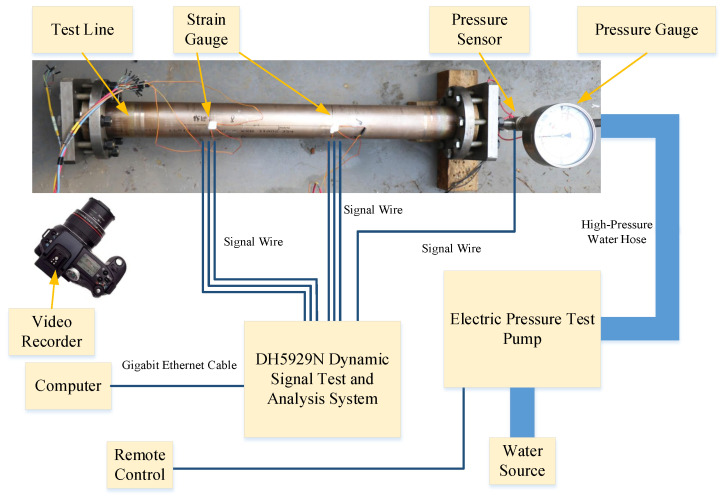
Schematic diagram of the test equipment connection.

Hydrostatic burst tests in this study were conducted in accordance with the GB/T 241-2007 “Metal tube hydrostatic test method” [29]. The test system consists of two main modules: a pressure application module and a measurement module. A schematic of the test setup is shown in Figure 3. An electric pressure test pump is connected to the sample through high-pressure hoses. The internal pressure of the sample requires real-time monitoring. Owing to the high testing pressures, a pressure gauge was installed in addition to the pressure sensor between the water outlet and the high-pressure hose to provide redundant measurements and verification. The pressure sensor has a range of 0–60 MPa with a 0–5 V output. Given the high accuracy of the pressure sensor compared with the pressure gauge (which can be read only on-site or from recorded video with relatively lower precision), the experimental results are based on pressure sensor readings.

This test simulates the process of pipeline bulging until failure under pressurized conditions in seawater pipelines to validate the accuracy of the modified ultimate load capacity assessment model. The ultimate limit state of the load-bearing capacity of a seawater pipeline is characterized by bursting or through-wall leakage. Therefore, the ultimate load capacity of the seawater pipeline corresponds to its burst pressure. The dimensional parameters of the Cu-Ni alloy seawater pipelines used in the tests are presented in Table 4.

## 3. Results and Discussion

### 3.1. Finite Element Simulation of Failure Characteristics in Seawater Pipelines with Double Pitting Defects

**Figure 4:**
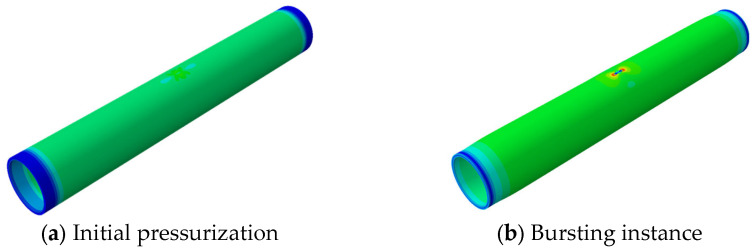
Blast Morphology of Double Pitting Corrosion Defects.

**Figure 5:**
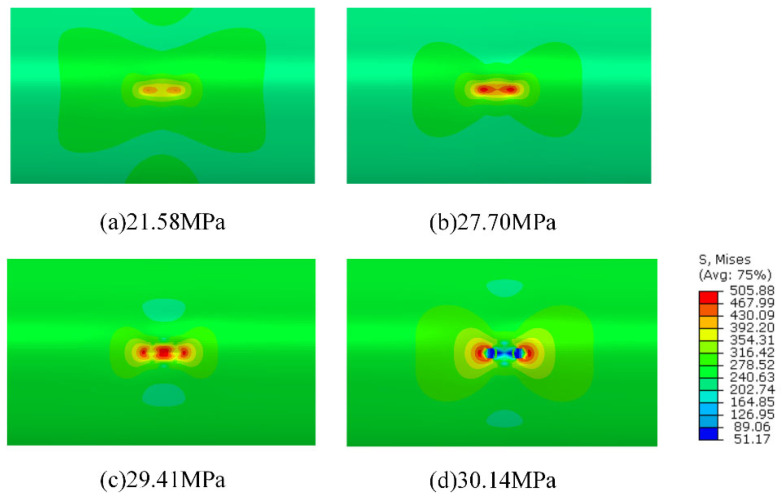
Von Mises stress nephogram of pipeline AP8# under different internal pressures.

**Figure 12:**
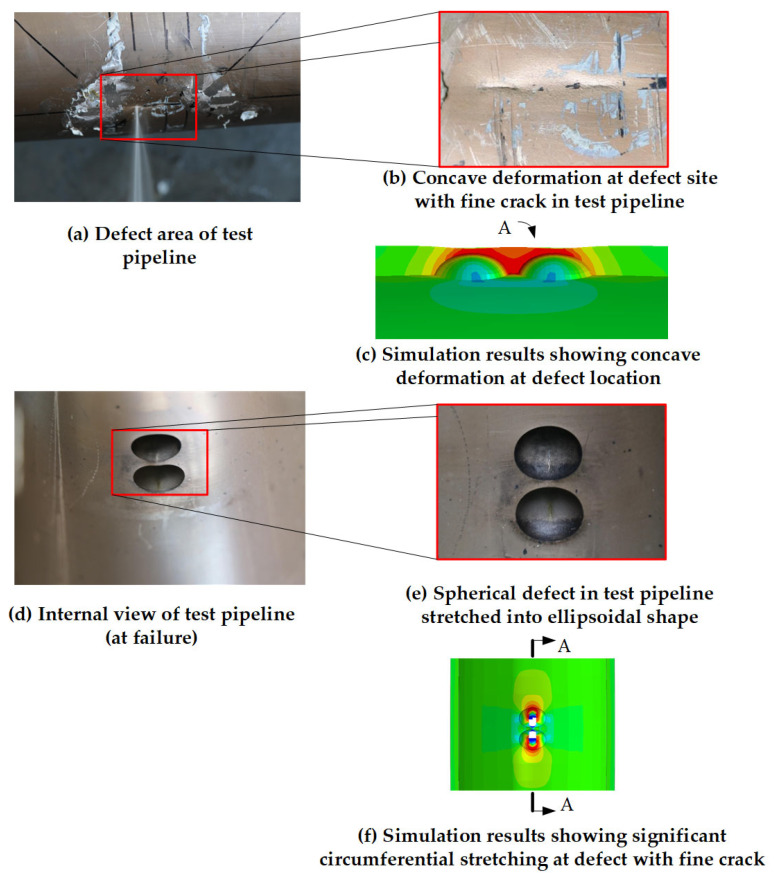
Comparison of fracture morphology. (Figure (c) presents a cross-section of Figure (f) at A-A, rotated 90° clockwise).

In accordance with the pipeline parameters provided in Table 2, numerical simulations were conducted, and the corresponding results were obtained. The von Mises stress distribution is presented as color nephograms in Figure 4, Figure 5 and Figure 12c,f, where the blue-to-red gradient indicates increasing stress (see scale in Figure 5).The isometric views of pipeline AP8# (from Table 2) at two critical stages are shown in Figure 4: the initial pressurization and the moment of burst. As shown in Figure 4b, the pipeline exhibits significant bulging under internal pressure, whereas no obvious swelling is observed at the defect locations.

Figure 5 presents cloud diagrams depicting the bursting process in the defect region of pipeline AP8# under internal pressure. With increasing pressure, significant stress concentration develops at the midpoint between the two defects and in the intermediate connecting region, confirming their interaction along the centerline. Elements within both the defect areas and this intermediate region sequentially undergo elastic deformation, plastic flow, and ultimately failure.

Figure 5c shows the stress contour contracting simultaneously from both defect locations toward the midpoint, indicating the onset of material softening or failure in those elements. This stress relaxation initiates in the central region between the defects. By Figure 5d, the degradation has fully propagated through the wall thickness at the defect sites. Subsequent necking leads to pipeline depressurization and the formation of axial cracks. Whether these cracks from the two defects coalesce in the central junction region following depressurization cannot be conclusively determined from this simulation.

### 3.2. Influence of Defect Parameters on the Ultimate Load Capacity

#### 3.2.1. Influence of Defect Parameters on the Ultimate Load Capacity Under Constant Corrosion Diameter

**Figure 6:**
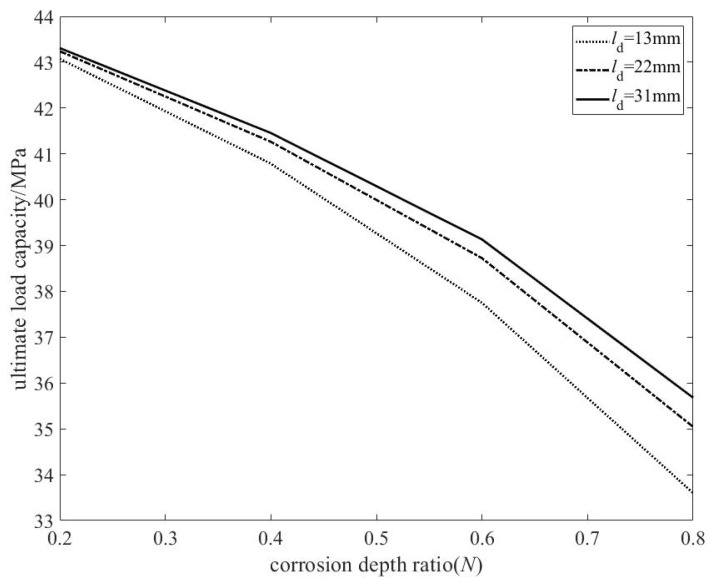
Relationship between double pitting defect depth and ultimate load capacity.

Based on the ultimate load capacity data from the 28 pipelines in Table 2, the relationship between the corrosion defect depth ratio *N* and the ultimate load capacity was derived, as shown in Figure 6.

① Overall trend: The ultimate load capacity exhibits a significant negative correlation with the defect depth ratio *N*. As *N* increases from 0.2 to 0.8, the ultimate load capacity decreases by approximately 10 MPa (about a 20% reduction), indicating that material loss has a decisive impact on bearing capacity.
② Nonlinear acceleration effect: The slope of the curve increases noticeably with higher *N* values, demonstrating a nonlinear amplification effect of defect depth. When *N* < 0.4, the reduction in bearing capacity is gradual (approximately 0.5 MPa decrease per 0.1 increase in depth ratio). However, when *N* > 0.5, the decline accelerates sharply (exceeding 1 MPa decrease per 0.1 increase in depth ratio). This suggests that the structure enters a sensitive instability range once the defect depth exceeds half of the wall thickness.

The nonlinear relationship between defect depth and ultimate load capacity arises because, when *N* is relatively small, the defect only induces localized stress concentration, with the plastic zone confined to the vicinity of the defect. Under such conditions, the overall cross-section remains capable of effectively carrying the load. In contrast, when *N* is larger (particularly exceeding 50% of the wall thickness), the defect leads to a comprehensive reduction in both the stiffness and strength of the cross-section. Furthermore, it triggers a complex coupling between local bending and membrane stresses, driving the structure into a “defect-sensitive zone” and resulting in an accelerated decline in load-bearing capacity.

**Figure 7:**
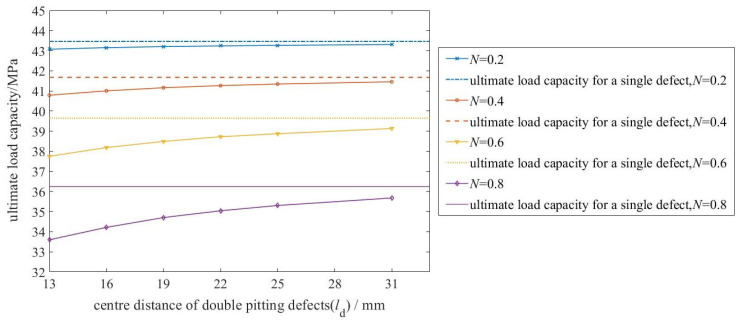
Relationship between the center distance of double pitting defects and the ultimate load capacity (for different corrosion depth ratios *N*).

Similarly, based on the simulation results of the pipelines in Table 2, the relationship between the center distance of the two defects and the ultimate load capacity was derived, as shown in Figure 7.

The analysis reveals the following:

① The ultimate load capacity increases monotonically with the defect center distance *l*_d_, which aligns with the physical intuition that “the interaction between dual defects weakens with distance.”
② Nonlinearity and saturation of spacing influence: Within the range of *l*_d_ < 20 mm, the load capacity recovers rapidly as the spacing increases (with a steeper gradient). When *l*_d_ > 25 mm, the curve tends to flatten, indicating that the stress interference between the dual defects has diminished to a negligible level, and the structural behavior approximates that of an isolated defect.

Further analysis reveals the coupling effect between depth and spacing:

① For the deep defect (*N* = 0.8) curve: within the range of *l*_d_ < 20 mm, the curve exhibits a steeper ascending slope, indicating that the interaction between deep defects is stronger and that a larger spacing is required for the load-bearing capacity to recover to the level of a single pit defect.
② For the shallow defect (*N* = 0.2) curve: the overall variation is mild, and even at small spacings, its load-bearing capacity is already close to the value of a single pit defect, suggesting that the interaction between shallow defects is weaker.

The observed relationship between the center distance of double defects and the ultimate load capacity can be attributed to the following mechanisms: under small spacing conditions (*l*_d_ < 1.5 times the defect diameter), the stress fields of the two defects strongly overlap, forming a joint weakening zone that significantly reduces the effective load-bearing cross-sectional area. As the spacing increases, the stress fields gradually separate, and the interference effect weakens accordingly, allowing the load capacity to recover toward the level of a single pit defect.

Furthermore, depth significantly amplifies the interference effect: deep defects cause broader and more intense disturbances to the stress field, leading to a wider range of interaction and more pronounced effects. This is reflected in the steeper slope of the deep defect curve within the small-to-medium spacing range and the longer distance required for convergence to the load capacity of a single pit defect.

When the spacing is sufficiently large (approximately *l*_d_ > 30 mm), the load capacity converges to the corresponding value of a single pit defect regardless of the defect depth. This indicates that the interaction between double defects has a well-defined decay scale, providing a solid physical basis for the “distance influence function” proposed in this study.

#### 3.2.2. Influence of Defect Parameters on the Ultimate Load Capacity Under a Constant Corrosion Depth Ratio

**Figure 8:**
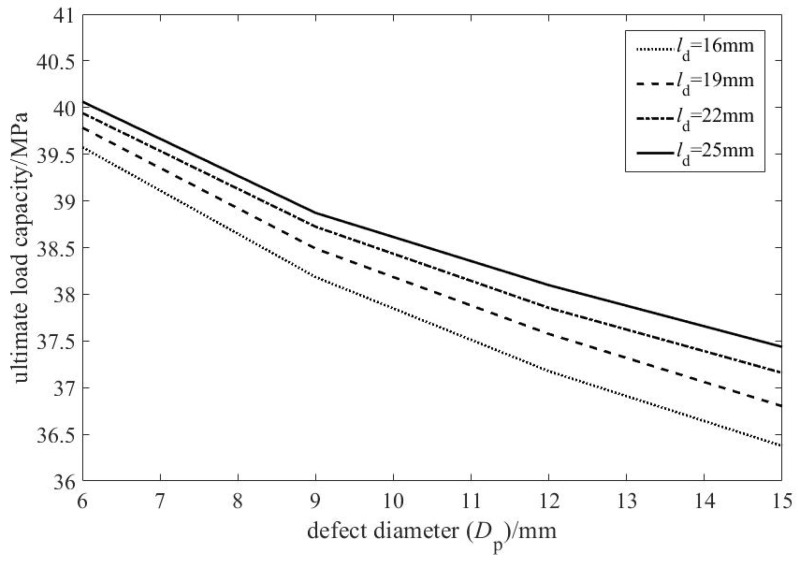
Relationship between double pitting defect diameter and ultimate load capacity.

Figure 8 presents the relationship between defect diameter and ultimate load capacity, based on simulation results for the 28 pipelines listed in Table 3.

Overall, the ultimate load capacity decreases with increasing defect diameter, confirming a negative correlation. Unlike the effect of depth ratio, however, the slope of this curve flattens as the diameter grows. This indicates a saturation effect: the detrimental influence of defect size on capacity diminishes at larger diameters.

This behavior stems from the evolution of stress states. Small defects create severe local stress concentration, causing a pronounced initial drop in capacity. As the defect enlarges, stress distribution within it becomes more uniform, and the stress concentration factor increases at a diminishing rate. Concurrently, stress redistribution in the remaining sound ligament of the pipe becomes more effective. Together, these mechanisms progressively mitigate the rate of capacity reduction.

**Figure 9:**
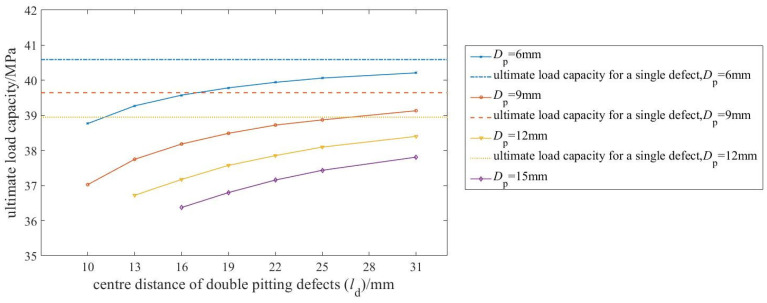
Relationship between center distance of double pitting defects and the ultimate load capacity (for different corrosion diameters $D_{P}$).

Figure 9 shows the relationship between the center-to-center distance of the two defects and the ultimate load capacity.

As shown in Figure 9, the ultimate load capacity increases nonlinearly with the center-to-center distance (from 10 to 31 mm). The increase is steep at small spacings (e.g., 10–16 mm) but decelerates significantly beyond approximately 22 mm, asymptotically approaching a stable plateau. This plateau value corresponds to the ultimate load capacity of a pipeline with a single, equivalent defect defined by the parameters of the double pitting defects.

From the perspective of the influencing mechanism, the change in the distance between defect centers essentially reflects the degree of interaction between the stress fields of the two corroded regions. When the distance between the centers is small, the stress concentration zones of the two defects overlap, resulting in stronger stress interference and a significant reduction in the effective load-bearing area of the material. As the distance between the centers increases, the overlap between the stress fields of the two defects decreases, the mutual interference weakens, and the effective load-bearing area of the pipeline gradually recovers.

Further analysis indicates that the influence of defect spacing on load-bearing capacity is more pronounced for larger-diameter defects. This heightened sensitivity stems from their broader inherent stress concentration zones. At small spacings, the extensive overlap of these zones produces strong synergistic weakening, causing a marked drop in capacity. Conversely, as spacing increases, the pronounced separation of the stress fields enables a more substantial recovery. Consequently, for larger defects, the deviation in capacity from the equivalent single-defect case exhibits greater variation with changing spacing.

### 3.3. Ultimate Load Capacity Model for Cu-Ni Alloy Pipelines with Double Pitting Defects

#### 3.3.1. Modification of the Ultimate Load Capacity Model

To address the poor agreement between the predicted results and finite element results for axial double-groove corrosion defects reported in the literature [16], this study attempted to establish an ultimate load capacity assessment model for pipelines with double defects by fitting a nonlinear function to characterize the relationship between the interdefect center distance and ultimate load capacity.

Building on the previously established influence of defect parameters, this study proposes a model for the ultimate load capacity of pipelines with two axially aligned, identical pitting defects. The model leverages the single-defect capacity as a baseline. When the defects are sufficiently separated, their interaction vanishes, and the double-defect capacity converges to this baseline value. At the opposite extreme, when defects are adjacent but not overlapping, the interaction is strongest. The effect of center distance varies monotonically between these bounds. This continuous influence is captured by a distance-dependent function, termed the distance influence factor w. Thus, the ultimate load capacity for axial double pitting defects is formulated as the product of w and the ultimate load capacity of the corresponding single-defect pipeline.

For thick-walled seawater pipelines containing single pit defects, reference [30] provides a relatively accurate calculation method:

(1) $$\left\{\begin{array}{l} p_{CP}=p_{0}\cdot R=p_{0}\cdot \left(1.0556\cdot \frac{1-0.785N}{1-0.785N\cdot Q_{P}^{-1}}\right)\\ p_{0}=\left[\frac{1}{2}(1+\eta^{2})-\frac{1}{3}\eta^{3}\right]\frac{2\sqrt{3}t\cdot \sigma_{b}}{D-t}\\ Q_{P}={\left[1.1834+\frac{L_{Z}^{2}}{D\cdot t}\right]}^{1/2} \end{array}\right.$$

In the above equation, $p_{CP}$ denotes the ultimate load capacity of the Cu-Ni alloy seawater pipeline with a single pit defect.

The distance influence factor $w(l_{d})$ must be a continuous function, where $0<w(l_{d})<1$. It attains its minimum value at the smallest center distance and asymptotically approaches unity as the distance increases. Among common functions, the exponential form is well-suited due to its inherent convergent behavior and flexibility. Therefore, an exponential function is adopted to meet these criteria, yielding the following expression for:

(2) $$w(l_{d})=-e^{-l_{d}}+1$$

Direct application of Equation (2) leads to excessive deviation, necessitating further modification. Figure 6 shows that the influence of center distance on ultimate load capacity strengthens with defect depth, indicating that the depth parameter must be integrated into the distance influence factor. We assume a linear influence of depth at a fixed spacing and that the spacing-capacity correlation is not purely exponential. To address this, a scaling coefficient is applied to the center distance parameter. Consequently, the distance influence factor is defined as follows:

(3) $$w(l_{d},N)=-e^{-(x_{3}\cdot l_{d}+x_{1}N+x_{2})}+1$$

The optimization objective is to minimize the sum of squared errors, *e*(*r*), between the finite element simulation results and the values predicted by the modified formula.

(4) $$e(r)=\sum_{n=1}^{49}(p_{FEA}-w(l_{d},N)\cdot p_{Eq.(1)}{)}^{2}$$

In the above equation, $p_{FEA}$ represents the finite element simulation results for the pipelines numbered in Table 2 and Table 3.

Using the least squares method with $x={\left[x_{1},x_{2},x_{3}\right]}^{T}$ as the design variable, the optimal fit is obtained when the value of the error function *e*(*r*) is minimized. The optimal design variables were found to be $x={\left[-0.95,\;2.062\;0.101\right]}^{T}$. On the basis of these results, the modified formula can be written as follows:

(5) $$\left\{\begin{array}{l} p_{CAP}=w(l_{d},N)\cdot p_{0}\cdot R=w(l_{d},N)\cdot p_{0}\cdot \left(1.0556\cdot \frac{1-0.785N}{1-0.785N\cdot Q_{P}^{-1}}\right)\\ w(l_{d},N)=-e^{-(0.101l_{d}-0.95N+2.062)}+1\\ p_{0}=\left[\frac{1}{2}(1+\eta^{2})-\frac{1}{3}\eta^{3}\right]\frac{2\sqrt{3}t\cdot \sigma_{b}}{D-t}\\ Q_{P}={\left[1.1834+\frac{L_{Z}^{2}}{D\cdot t}\right]}^{1/2} \end{array}\right.$$

In the above equation, $p_{CAP}$ represents the ultimate load capacity of the seawater pipeline made of Cu-Ni alloy material containing double pitting defects.

**Figure 10:**
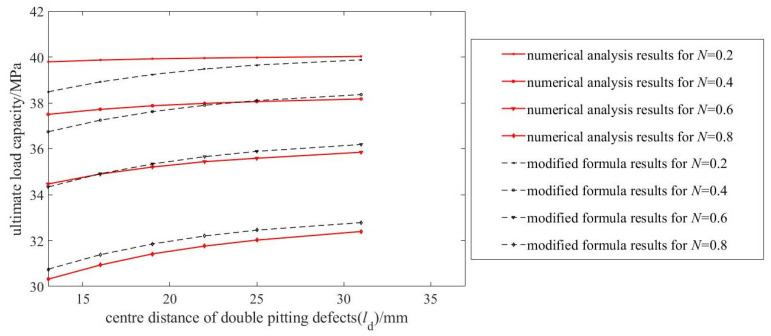
Correction effectiveness of the modified formula for double pitting defects.

Figure 10 compares the predictions of the modified formula with simulation results for pipelines containing double pitting defects of varying depths, based on 49 sets of ultimate load capacity data. The modified formula demonstrates higher prediction accuracy for deeper defects, and for shallower defects with small center distances, the prediction error remains below 1 MPa.

Applying the results of Equation (5) to predict the burst pressure of test pipelines, and substituting the pipeline parameters from Table 4, the predicted burst pressure for Test-1# was 29.131 MPa, and that for Test-2# was 30.176 MPa.

#### 3.3.2. Experimental Validation of the Modified Ultimate Load Capacity Model

**Figure 11:**
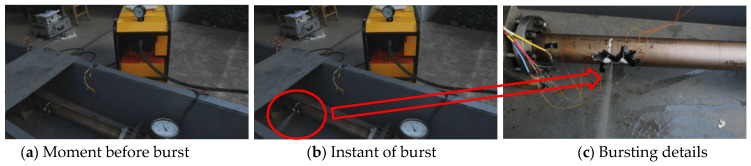
Documentation of the bursting process in pipeline 1#.

To validate the accuracy of the modified ultimate bearing capacity model, experimental verification was conducted to assess its predictive performance. In order to mitigate the influence of random factors during testing and to ensure the accuracy of measured data as well as the reliability of the results, two repeated tests were carried out. Following the experimental scheme described previously, two hydrostatic burst tests were conducted on pipelines containing double pitting defects with equal defect diameter and depth but different center distances. The pipelines were designated Test-1# and Test-2#, and their parameters are detailed in Table 1. Both tests successfully resulted in failure at predetermined defect locations. A comparative sequence of moments immediately before and during the bursting instance from one of the tests is shown in Figure 11. The circled area in (b) provides an overview of the water jet upon burst, while the arrow in (c) indicates details of water ejection from the opening.

Pipeline failure occurred with minimal acoustic emission, producing only a fine, low-impact water jet upon rupture, characteristic of through-wall pressure relief. While the pressure test pump remained operational, residual pressure was maintained within the pipeline system. After the test pump was deactivated, the internal water pressure decreased rapidly.

On the basis of the experimental measurements, the burst pressure for Test-1# was determined to be 28.356 MPa, whereas that for Test-2# was 29.736 MPa.

#### 3.3.3. Analysis of Simulation and Experimental Results

##### Comparative Analysis of Ultimate Load Capacity

Based on explicit finite element results, this study introduces a distance influence factor $w(l_{d},N)$ to characterize the relationship between interdefect center distance and ultimate load capacity. Using these results, a corresponding load capacity model for Cu-Ni alloy seawater pipelines with double pitting defects was developed via curve fitting.

As shown in Table 5, the double pitting defect ultimate load capacity model proposed in this study demonstrates high accuracy in predicting the burst pressure of pipelines with double defects. The maximum relative error is only 2.73%, indicating excellent agreement between the predicted results and experimental values.

Apart from random errors introduced by the measurement process itself, the discrepancies in the results of this study primarily stem from two factors. First, there is the simplification in the material constitutive model. The numerical simulation employed an ideal elastic-plastic constitutive model derived from standard tensile tests. However, the actual mechanical response of the copper-nickel alloy under high pressure and complex multiaxial stress states is more intricate, and the material may exhibit slight anisotropic characteristics. Second, there are limitations in the machining accuracy of the actual defects. The double pitting corrosion defects on the inner wall of the pipeline were fabricated using electrical discharge machining. Although the machining error can be controlled within ±0.1 mm, subtle deviations between the actual defect geometry and the idealized geometry assumed in the numerical model cannot be entirely eliminated.

##### Comparative Analysis of Failure Characteristics

Pipeline 1# burst at the expected location. Observations of the post-failure morphology at the defect sites reveal significant circumferential bulging around the defects, with noticeable wall thinning and the formation of two small pits at the defect locations. A fine linear crack appeared along the pipe axis, with a length approximately equal to the defect size. Notably, both defect locations exhibited substantial plastic deformation. While leakage occurred from the left defect, the right defect, although not ruptured, also experienced severe plastic deformation.

Figure 12 presents a comparison between the simulation and experimental results. From the experimental perspective, Figure 12b shows that the copper-nickel alloy pipeline exhibited pronounced inward-buckling plastic instability at the defect location, with the fracture edges displaying characteristic ductile thinning. Figure 12d,e further reveals that the internal structure underwent tensile deformation without brittle fracture, which is consistent with the inherent high ductility of copper-nickel alloys. From the numerical prediction side, the simulation results in Figure 12c,f (based on the true stress–strain curve of the copper-nickel alloy) accurately predicted failure at the same location and reproduced the entire process of continuous plastic strain accumulation, wall-thinning, and structural inward collapse. This validates the model’s capability to capture the dominant failure mechanism of copper-nickel alloy pipelines, i.e., ductile failure governed by plastic instability.

## 4. Conclusions

This study examined the ultimate load capacity of Cu-Ni alloy pipelines with double pitting defects. The results demonstrate that at a fixed depth, increasing defect diameter reduces capacity, but this effect attenuates nonlinearly with diameter. Furthermore, a nonlinear positive correlation exists between defect spacing and capacity, a relationship governed by defect diameter: larger diameters amplify the influence of spacing variation. These findings provide a valuable basis for assessing the residual strength of corroded pipelines in marine and petrochemical systems.

The presented model is specific to axially aligned double pitting defects under quasi-static internal pressure. To broaden its applicability, future work should: (1) expand to varied defect geometries and distributions, (2) apply the methodology to other alloys, and (3) incorporate combined loads (axial, bending, cyclic, impact) to simulate real-world service conditions.

## Figures and Tables

**Table 1:** Material properties of Cu-Ni alloy.

Parameter	Value
Tensile Strength	306 MPa
Yield Strength	120 MPa
Elongation	32.5%
Poisson’s Ratio	0.33
Young’s Modulus	136 GPa
Density	8.94 g/cm^3^

**Table 2:** Numbering of pipelines with axial double pitting defects (for different corrosion depth ratios *N*).

*l_d_*	*N* = 0.2	*N* = 0.4	*N* = 0.6	*N* = 0.8
*l*_d_ = 13 mm	AP1#	AP2#	AP3#	AP4#
*l*_d_ = 16 mm	AP5#	AP6#	AP7#	AP8#
*l*_d_ = 19 mm	AP9#	AP10#	AP11#	AP12#
*l*_d_ = 22 mm	AP13#	AP14#	AP15#	AP16#
*l*_d_ = 25 mm	AP17#	AP18#	AP19#	AP20#
*l*_d_ = 31 mm	AP21#	AP22#	AP23#	AP24#
*l*_d_ = 37 mm	AP25#	AP26#	AP27#	AP28#

Note: the hash symbol (#) demotes “No.” (i.e., AP1# stands for test pipeline No.1). The same meaning applies to the # symbol in Table 3 and Table 4.

**Table 3:** Numbering of pipelines with axial double pitting defects (for different corrosion diameters $D_{P}$).

*l_d_*	*D_p_* = 6	*D_p_* = 9	*D_p_* = 12	*D_p_* = 15
*l*_d_ = 13 mm	AP29#	AP30#	AP4#	AP31#
*l*_d_ = 16 mm	AP32#	AP33#	AP8#	AP34#
*l*_d_ = 19 mm	AP35#	AP36#	AP12#	AP37#
*l*_d_ = 22 mm	AP38#	AP39#	AP16#	AP40#
*l*_d_ = 25 mm	AP41#	AP42#	AP20#	AP43#
*l*_d_ = 31 mm	AP44#	AP45#	AP24#	AP46#
*l*_d_ = 37 mm	AP47#	AP48#	AP28#	AP49#

**Table 4:** Dimensional data of the seawater pipeline.

Pipeline No.	$D_{P}$	d	D	$D_{i}$	*t*	*l*_d_
Test-1#	12 mm	4.0 mm	90.0 mm	80.0 mm	5.0 mm	13.0 mm
Test-2#	12 mm	4.0 mm	90.0 mm	80.0 mm	5.0 mm	19.0 mm

Where $D_{P}$ is the defect diameter; d is the defect depth; *D* is the outer diameter of the pipe; *D*ᵢ is the inner diameter of the pipe; *t* is the wall thickness; $l_{d}$ is the center distance between defects.

**Table 5:** Comparison of Burst Test Results for Pipelines with Axial Double Pitting Defects (MPa).

Pipeline No.	Calculated Result (Equation (5))	Experimental Result	Absolute Error (MPa) (Experimental vs. Equation (5))	Relative Error (%) (Experimental vs. Equation (5))
Test-1#	29.131	28.356	0.775	2.73%
Test-2#	30.176	29.736	0.440	1.47%

## Data Availability

The original contributions presented in this study are included in the article. Further inquiries can be directed to the corresponding authors.
